# Degradation Characteristics of Microstructure and Mechanical Properties on the Cross-Section of a Massive Casting Made of G17Mn5 Steel

**DOI:** 10.3390/ma18163877

**Published:** 2025-08-19

**Authors:** Barbara Elżbieta Kalandyk, Dariusz Boroński, Paweł Maćkowiak, Małgorzata Trepczyńska-Łent, Justyna Kasińska, Sebastian Sobula

**Affiliations:** 1 Faculty of Foundry Engineering, AGH University of Krakow, 30 Mickiewicza Ave., 30-059 Krakow, Poland; sobula@agh.edu.pl; 2 Faculty of Mechanical Engineering, Bydgoszcz University of Science and Technology, Al. Prof. S. Kaliskiego 7, 85-796 Bydgoszcz, Poland; dariusz.boronski@pbs.edu.pl (D.B.); pawel.mackowiak@pbs.edu.pl (P.M.); malgorzata.trepczynsla-lent@pbs.edu.pl (M.T.-Ł.); 3 Faculty of Mechatronics and Mechanical Engineering, Kielce University of Technology, 7-Tysiąclecia Państwa Polskiego Ave., 25-314 Kielce, Poland; kasinska@tu.kielce.pl

**Keywords:** slag ladle, low-carbon cast steel, microstructure, mechanical properties, fractography

## Abstract

This paper presents the changes in microstructure and mechanical properties that occurred across the wall cross-section of a massive slag ladle casting due to service conditions. The slag ladle was made of low-carbon cast steel. Based on the test results, it was shown that the working environment influenced the macro-segregation of C and S on the cross-section of the wall and, consequently, had an effect on the changes in microstructure. A pearlitic–ferritic microstructure was found in the central part, while in the outer and inner parts of the wall, the microstructure was of a ferritic–pearlitic type. This change mainly influenced the impact energy—the lowest values were obtained at the centre of the wall (24 J at +20 °C). In the remaining areas tested on the wall cross-section at +20 °C, the impact energy exceeded the minimum required value of 27 J in the Charpy test. The tests revealed the presence of a network of cracks in areas adjacent to the inner surface of the ladle wall, which had a negative impact on the impact energy values, as did the presence of non-metallic inclusions. The changes found in the microstructure as a result of the ladle operation caused significant differences in properties such as impact energy and hardness, while also affecting, though to a lesser extent, the mechanical properties (UTS = 397–434 MPa; YS = 222–236 MPa).

## 1. Introduction

Slag ladles are castings used to collect and transport liquid slag from metallurgical processes such as steelmaking or the extraction of other metals. Slag ladle castings are made of low-carbon steel (PN-EN 10293:2015 [1], e.g., G17Mn5 or ASTM A27 grade 60–30, and ASTM A27/A27M-2020 [2]) or ductile iron (PN-EN 1563: 2000) [3]. These alloys are characterised by a yield strength (YS) > 205 MPa, an ultimate tensile strength (UTS) = 405–600 MPa, and an elongation (EL) > 24% [1,2,3,4]. The castings are produced in a wide range of dimensions from small to large. Ladles with a capacity of 16–18 m^3^ are classified as massive (large-size) castings, and, therefore, their production technology causes many problems, not only in terms of liquid metal preparation but also in terms of the moulding technology and casting solidification conditions. For this reason, the technological solutions introduced mainly concern innovations in the melt processing technique [5,6,7]. Enhancing the metallurgical cleanliness of liquid steel is crucial in this context. The primary objective of these processes is to reduce the concentration of non-metallic inclusions, with particular emphasis on continuous sulphides and oxides located at grain boundaries, classified as type II [8]. The harmful effect of non-metallic inclusions depends not only on their quantity but also on their shape, size, and distribution. These inclusions are particularly dangerous in castings due to their significant impact on reducing plasticity. In steels (as-cast), the characteristics of MnS inclusions are significantly affected by elemental segregation, solidification conditions, and the cooling rate during casting [9]. An important problem in the operation of such castings is the effect of thermal shocks to which they are exposed. Thermal shocks arise as a result of the large difference in temperature between the ladle and the liquid slag, which changes its state during transport to the storage site. One of the consequences is the high thermal stresses that occur in these castings and are accompanied by, among other phenomena, plastic deformation, which is responsible for alloy degradation and thermal fatigue [9,10]. These stresses result in cracks, which most often appear in the lower part of massive slag ladle castings made of low-carbon steel. The authors of [9] report that with the increasing number of filling and unloading cycles, as well as changes in ambient temperature (summer/winter), the service life of steel slag ladles varies within wide limits, i.e., from 500 to even 1000 cycles. The quality and service life of steel castings significantly depend on the melt purity and the type of heat treatment to which the finished ladle castings are subjected [10,11,12]. Massive steel slag ladles are heat-treated after casting to ensure a uniform ferritic–pearlitic microstructure on the entire wall cross-section. For this purpose, after austenitizing, controlled cooling is used with a pause at a temperature of approx. 600–630 °C, followed by further cooling in the furnace to 300 °C and cooling of the castings in air. This type of casting heat treatment provides the following mechanical properties: UTS = 472–496 MPa; YS = 300 MPa; EL = 33–37%; and impact energy = 70 J at room temperature [1].

This article presents the results of tests carried out on the cross-section of the wall of a steel ladle with a capacity of 16–18 m^3^ withdrawn from use, with particular attention paid to the changes that occurred in the microstructure and selected mechanical properties on the cross-section of the casting wall, with an approx. 130 mm thickness. The mechanical test results show that the prolonged exposure of low-carbon steel casting to high-temperature service conditions influences its mechanical properties, particularly its impact strength. We found that in the region subjected to the highest temperatures, the impact energy significantly decreased below the minimum required value of 27 J in the Charpy impact test. This finding is not commonly reported in the scientific literature related to steel casting manufacturing.

The scope of this research also included the investigation of changes in impact strength at varying temperatures due to the non-uniform service conditions of slag ladles, such as seasonal differences between winter and summer. Impact strength and cracks caused by thermal fatigue are the key material properties determining the decision to withdraw a slag ladle from service. Our research complements existing knowledge in the field of mechanical property changes in slag ladles at the end of their service life and addresses aspects that research has not previously been published on. Therefore, this study makes a significant contribution to production techniques for slag ladles, particularly in terms of changes in their chemical composition and melting practices. It is well known that these factors influence metallurgical cleanliness, which in turn affects the impact strength, especially in temperatures below 0 °C.

## 2. Materials and Methods

**Figure 1:**
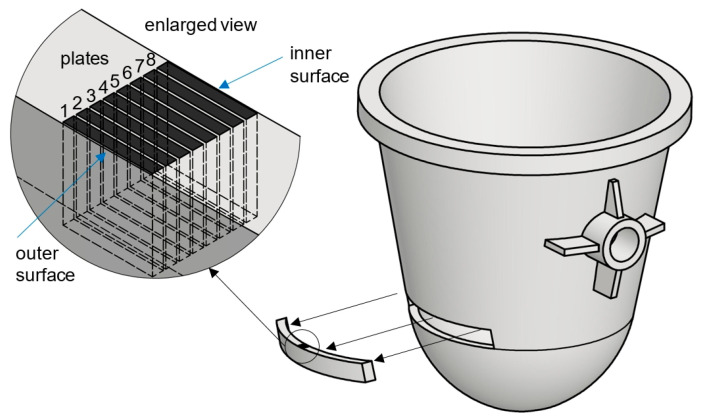
Slag ladle: view of a fragment of the casting wall with eight marked plates from which samples were cut.

**Figure 2:**
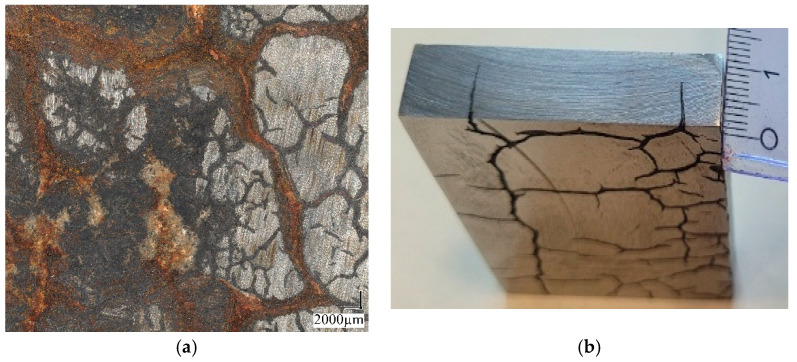
View of the inner surface of the ladle wall before and after grinding out irregularities (**a**) and the example of crack depth in the wall cross-section (**b**).

Tests were carried out on cast steel samples taken from a decommissioned massive slag ladle with a capacity of 16–18 m^3^. The test material was taken from the ladle wall with a thickness of approx. 0.13 m located on the side of the ladle above its bottom. The material was divided into eight equal cuboid plates, each with a thickness of approx. 13 mm (Figure 1). Since in the last plate, the one most distant from the ladle’s outer surface, a network of cracks was revealed during grinding (Figure 2), this plate was excluded from further testing. From each of the remaining plates, numbered 1–7 (with numbers running across the ladle wall cross-section from the outer surface toward the inner surface), samples were cut out for impact and tensile tests. The chemical composition of the cast steel was determined using a SPECTROMAXX spectrometer (Table 1). The chemical composition was also analysed in the outer, middle, and inner areas of the cross-section of the wall (Spectro Analytical Instruments, Kleve, Germany). Additional oxygen content measurements in the casting wall were performed using a LECO RO16 analyser (LECO Corp., St Joseph, Michigan, MI, USA) following the recommended procedure. The measurements were taken in accordance with the recommended procedure. Impact tests were performed using a Hoytom pendulum impact tester (KB Pruftechnik Gmbh, Hochdorf-Asseiheim, Germany) with a fracture energy capacity of 300 J in accordance with the Charpy impact testing standard [13] using 10 × 10 × 55 mm specimens with a 2 mm deep “V” notch (ISO 148-1:2010). The impact strength tests were performed at three temperatures: +20 °C, 0 °C, and −10 °C. The temperatures were selected based on the actual operating conditions of slag ladles during summer and winter. This refers to the transport of ladles with liquid slag from the hall to the metallurgical waste collection site located outdoors. The tests were designed to demonstrate the rate at which impact energy performance values decline with decreasing ambient temperature. Metallographic examinations were carried out using light and scanning microscopy. A Leica MEF 4M microscope equipped with a Leica DFC290 camera and a JEOL JSM 7100F scanning electron microscope (Akishima, Tokyo, Japan) with an EDS detector from Oxford Industries (Abingdon, Oxon, UK) were employed. Non-metallic inclusion measurements were performed using a Keyence VHX light microscope equipped with proprietary software. Each sample was examined over a surface area of 6.3 mm^2^ at a magnification of 400×.

**Figure 3:**
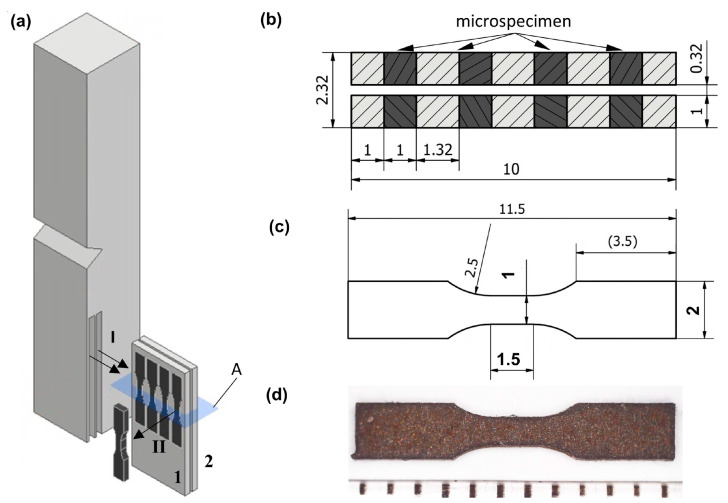
Microsample: (**a**) preparation; (**b**) cross-section in plane *A* through slices cut from the macroscopic sample, with microsample location marked on the drawing; (**c**) dimensions; (**d**) photograph of the microsample before testing.

Hardness was measured on the cross-section of the casting wall using a Nexus 7700G2 (Innovatest Europe BV, Maastricht, The Nederlands) hardness tester with a 2.5 mm diameter sintered carbide ball, a force of 187.5 kgf, and a dwell time of 15 s. Measurements were taken at 4 different sites on each plate (numbered 1–7) in accordance with the standard in [14]. Tensile tests were performed on microsamples taken from the macroscopic samples used for impact tests (Figure 3a). Static properties were determined on microsamples taken from five macroscopic samples, each representing one of the 7 plates on the wall cross-section. Initially, in Step I, two 1 mm thick strips were cut from each macroscopic sample and numbered 1 and 2, with a spacing of 0.32 mm between them (Figure 3b). Then, in Step II, microsamples were cut from these strips; their shapes and dimensions are shown in Figure 3c. Within a single strip, the spacing between adjacent working parts of the samples was 1.32 mm (Figure 3b). The wire electro-discharge machining (WEDM-BP95d, Kutno, Poland) method was used to cut the strips and obtain the final shape of the samples. For this purpose, a CuZn37 brass wire with a diameter of 0.25 mm was used. To minimise the thermal effect during the drilling process, a pulse current of 4.5 A, a voltage of 80 V, and an extended pause time between current pulses were applied, which also reduced the cutting speed. The remaining cutting parameters were selected based on those typically used for steel and cast iron. This method of sample preparation ensures that the microstructure undergoes only minimal changes.

**Figure 4:**
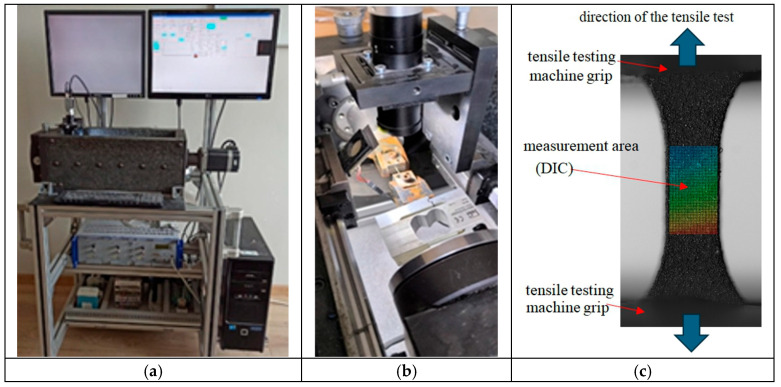
Test stand for determining mechanical properties—MFS system: (**a**) general view, (**b**) specimen zone during testing, (**c**) measurement of specimen deformation using digital image correlation.

To test the microsamples under static load conditions, the MFS fatigue testing microsystem (Micro Fatigue System, Bydgoszcz University of Science and Technology, Bydgoszcz, Poland) was employed. This system is dedicated to testing micro-objects and enables the application of forces via displacement generated by actuators. The displacement resolution covers the nanometre to micrometre range. The positioning precision of the nanoactuator was 1.7 nm, whereas that of the microactuator was 1 μm. Considering the dimensions of the sample measurement area, the digital image correlation (DIC) technique [15] was used to assess deformations. This allowed the measurement of deformations directly from the natural texture of the sample surface, thereby eliminating the need for additional markers. Visualisation of the tested sample area was obtained using high-class telecentric lenses from VS Technology (VS Technology Corporation, Ichikaba, Chiba, Japan), characterised by micrometre-level optical resolution, and high-resolution cameras from the Basler Ace series (acA4024-8gm, Basler AG, Ahrensburg, Germany). Figure 4 shows the general layout of the fatigue microsystem (Figure 4a) and an enlarged view of the sample area subjected to testing (Figure 4b).

## 3. Experimental Results

### 3.1. Changes in Microstructure Due to Operation

**Figure 5:**
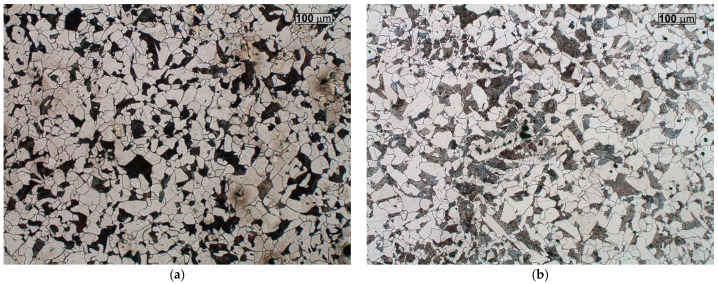
Microstructure of the tested cast steel on the outer surface of the ladle wall (plate 1) (**a**) and on the inner surface of the ladle wall (plate 7) (**b**); light microscopy, etched with Nital reagent.

**Figure 6:**
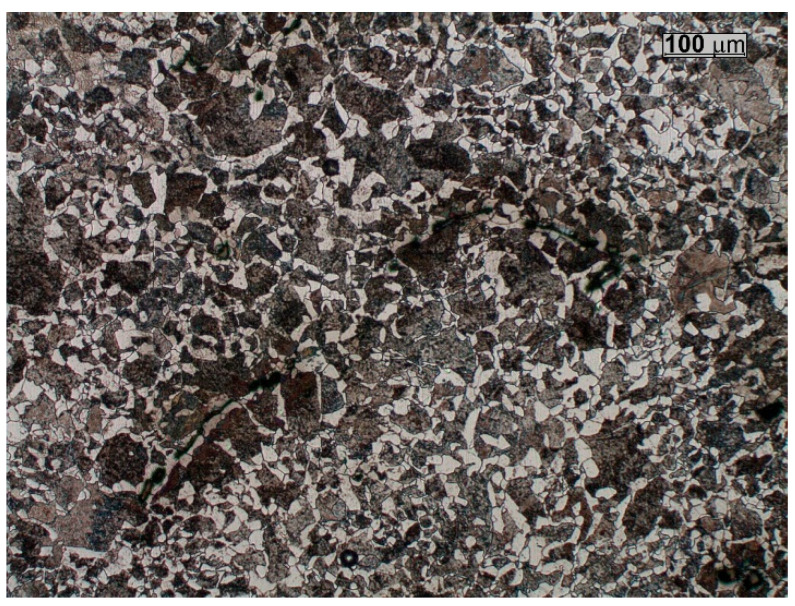
Microstructure of the tested cast steel in the centre of the ladle wall (plate 4); light microscopy, etched with Nital reagent.

The changes observed in the microstructure across the cross-section of the slag ladle wall reveal distinct differences between the centre of the wall and its other regions (Figure 5 and Figure 6). From the conducted studies, it follows that both outer and inner parts of the wall have a microstructure typical of low-carbon cast steel, i.e., the microstructure that consists mainly of ferrite and islands of pearlite (Figure 5). In the centre of the wall, the microstructure is of a pearlitic–ferritic type (Figure 6). Such changes in the microstructure are probably due to the cyclic heating and cooling processes to which the slag ladle is subjected during its operation. These processes lead to chemical segregation, primarily of C, across the cross-section of the wall. Sulphur is another element that strongly segregates in steel [4,16]. The spectral analysis carried out on the cross-section of the ladle wall confirmed an increase in carbon content to 0.25% C in the centre of the wall cross-section compared to other regions (0.19% C at the outer surface and 0.20% C at the inner surface of the casting wall, respectively). Sulphur was observed to undergo a similar process of segregation, reaching values of 0.03% S in the centre of the wall cross-section and approx. 0.014–0.015% S at the outer and inner surfaces, respectively. The analysis of oxygen content in the centre of the wall, as well as in the outer and inner regions, did not reveal any significant differences. The total oxygen content was high, ranging from 137 to 160 ppm (the recommended total oxygen content in the casting is approx. 40–60 ppm), and was therefore expected to promote the formation of inclusions of both type I and type II [8,9]. According to Jinlong et al. [17], oxygen content is not a direct factor influencing the morphology of MnS inclusions in low-oxygen steels, whereas larger amounts promote the formation of spherical, evenly distributed type I MnS inclusions.

On both the outer and inner wall surfaces, ductile ferrite is the primary microstructural constituent, while in the central region of the wall cross-section, hard pearlite is clearly predominant. This produces different mechanical properties across the wall thickness. Another important factor influencing the properties of the examined cast steel is the presence of non-metallic inclusions [7,18]. This results in different mechanical properties across the wall cross-section.

**Figure 7:**
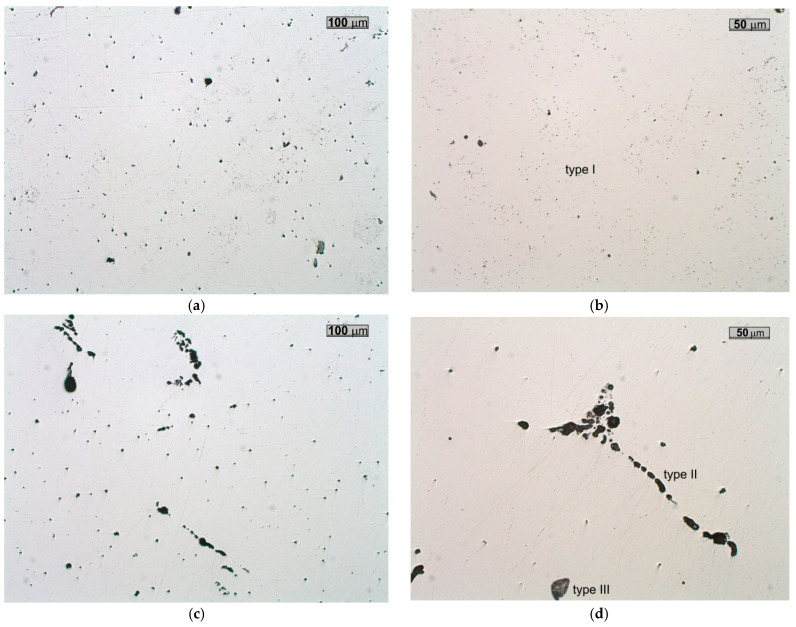
Characteristic forms of inclusions observed on the cross-section of the slag ladle wall: plate 1 (**a**,**b**), plate 7 (**c**,**d**); light microscope, unetched sections.

**Figure 8:**
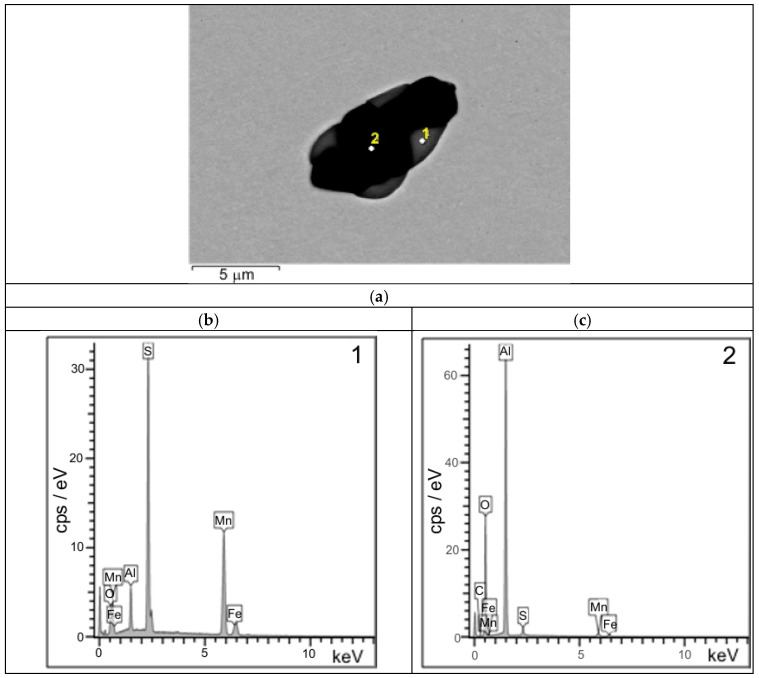
Example of SEM-EDS image of oxide-sulphide (**a**) with diffraction patterns from point 1 (**b**) and point 2 (**c**); scanning microscope.

**Figure 9:**
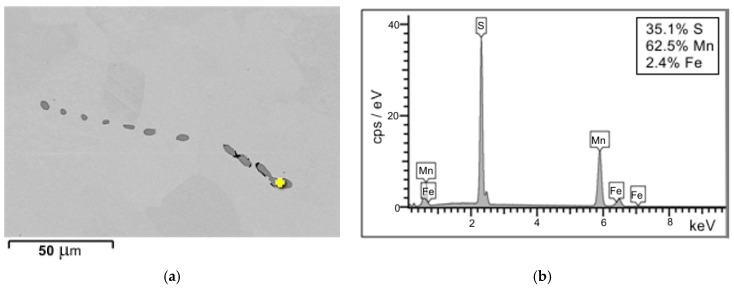
Two-dimensional SEM morphologies of type II sulphide inclusions (**a**) with diffraction pattern and chemical composition analysis of one MnS inclusion taken as an example (**b**); scanning electron microscope.

Examinations of unetched microsections reveal that, across the wall cross-section, inclusions of type I and type III, originating directly from the metal bath during the final stage of solidification, are predominant. These inclusions are mostly irregularly shaped oxide-sulphides (Figure 7a,b and Figure 8) [18,19,20]. In the region extending from the central zone of the ladle wall (plates 3 and 4) to its inner surface (plate 7), additional clusters of type II MnS inclusions, precipitating along the primary grain boundaries, were identified (Figure 7c,d). The distribution of type II sulphides is also favoured by the segregation of sulphur in the casting wall [20,21]. Type II MnS inclusions are a primary cause of reduced plasticity in the cast steel, including diminished impact strength (Figure 9) [12,21]. The only method that has a beneficial effect and can reduce the amount of non-metallic inclusions and change their morphology is the process of liquid steel refining, in which both the refining time and the introduced modifiers are of paramount importance [18,22,23]. By means of this metallurgical process, it is possible to control the size, shape, and distribution of non-metallic inclusions in castings and increase both cast steel toughness and impact strength [24]. In the case of massive castings, as in steels, the casting cooling and solidification rate is also an important parameter [25,26].

The non-metallic inclusion measurements presented in Table 2 indicate that the highest volume fraction was observed in plates 4 and 5, which were located in the central region of the slag ladle wall. This distribution of inclusions confirms the occurrence of elemental segregation during solidification of the slag ladle in the mould.

### 3.2. Hardness

**Figure 10:**
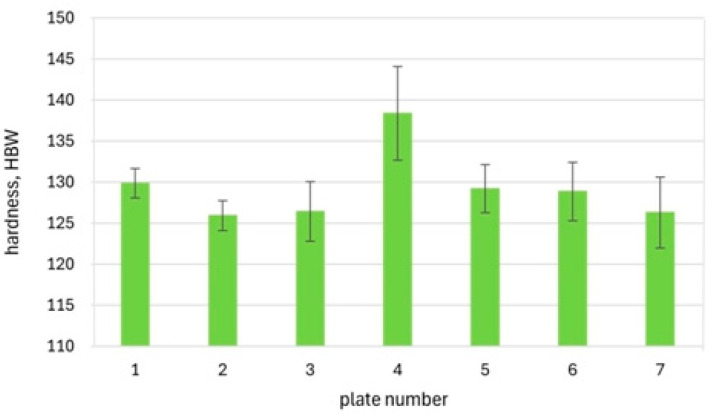
Hardness (HBW_10_) on the cross-section of the slag ladle wall.

Changes in the hardness of the tested cast steel, examined on the cross-section of the slag ladle wall, are shown in Figure 10.

The higher content of pearlite in the central part of the wall cross-section increased the hardness to approx. 138 HBW10. In the remaining areas of the wall cross-section, the hardness values were lower, ranging from 125 to 130 HBW10. Compared to the condition before the operation (after normalisation, the average hardness of the cast-on sample was 156 HBW10), the hardness after the operation decreased by approximately 20 to 30 HBW10. This reduction may result from differences in the solidification rate between the cast-on sample and the massive casting of the slag ladle (including differences in sample and casting mass, as well as wall thickness). Another possible factor is carbon segregation on a macroscopic scale.

### 3.3. Impact Energy

A significant reduction in the impact energy of the tested cast steel after operation was observed compared to the recommended value of 70 J [1].

**Figure 11:**
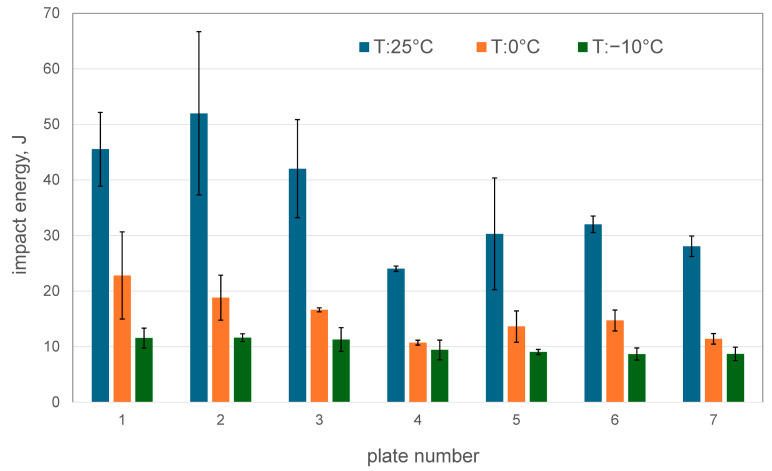
Impact energy on the cross-section of the wall thickness of the slag ladle casting at three test temperatures: +20 °C, 0 °C, and −10 °C.

Changes in the impact energy on the cross-section of the wall of the tested casting, measured at room temperature, 0 °C, and −10 °C, are shown in Figure 11. At room temperature, across the entire wall cross-section, the measured impact energy values exceed the brittleness threshold for cast steel (>27 J). However, throughout the tested area, lowering the test temperature to 0 °C and −10 °C causes a significant reduction in this value, to below 27 J. These low impact energy values indicate an increase in the brittleness of the slag ladle casting and may significantly influence its performance during winter operation. In this case, the requirement for the ductile-to-brittle transition temperature to be lower than the casting’s operating temperature was not met.

Tests of the impact energy at room temperature show that, on the wall cross-section, the highest values occur in the area adjacent to the outer surface of the ladle (42–53 J, plates 1, 2). As expected, in the central part of the wall, with pearlite predominance in the microstructure, low impact energy values were obtained (24 J; plate 4). These values are nearly two times lower than those measured for parts of the wall cross-section numbered 1 and 2. In turn, with a comparable microstructure, plates numbered 5–7 have an impact energy of 28–30 J. The decrease in impact energy in this part of the wall cross-section is due not only to the presence of non-metallic inclusions but also to the observed network of small cracks that penetrate into the test material of the ladle wall. Therefore, in addition to non-metallic inclusions and microcracks, the third factor responsible for the impact energy of the tested cast steel is macro-segregation within the cross-section of the casting wall.

**Figure 12:**
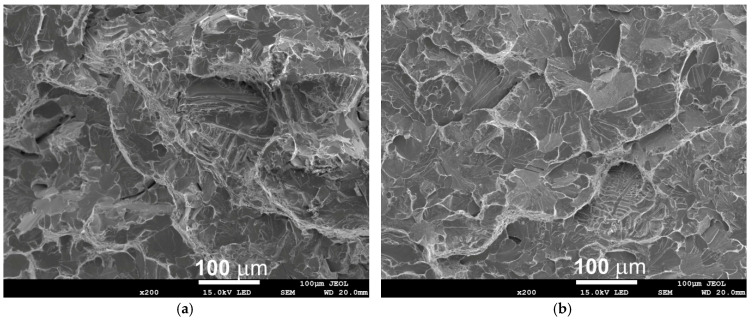
Example of the fracture surface in the centre of the wall at the test temperature of +20 °C (**a**,**b**).

The conducted fractographic analysis shows that the low values of the impact energy correspond to a mixed-mode fracture, with a predominance of brittle cleavage areas located in the central zone of the fracture surface, combined with the presence of microcracks (Figure 12). Figure 12b shows numerous variable fracture directions and faults in the cleavage plane. A visual assessment indicates that the fracture surfaces are flat, with small shear ridges occurring on the surface of the sample.

**Figure 13:**
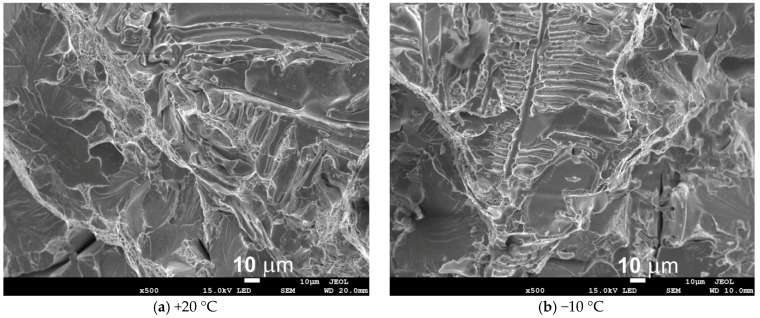
SEM images of the fracture surface from the centre of the wall at test temperatures of +20 °C (**a**) and −10 °C (**b**); scanning electron microscope.

**Figure 14:**
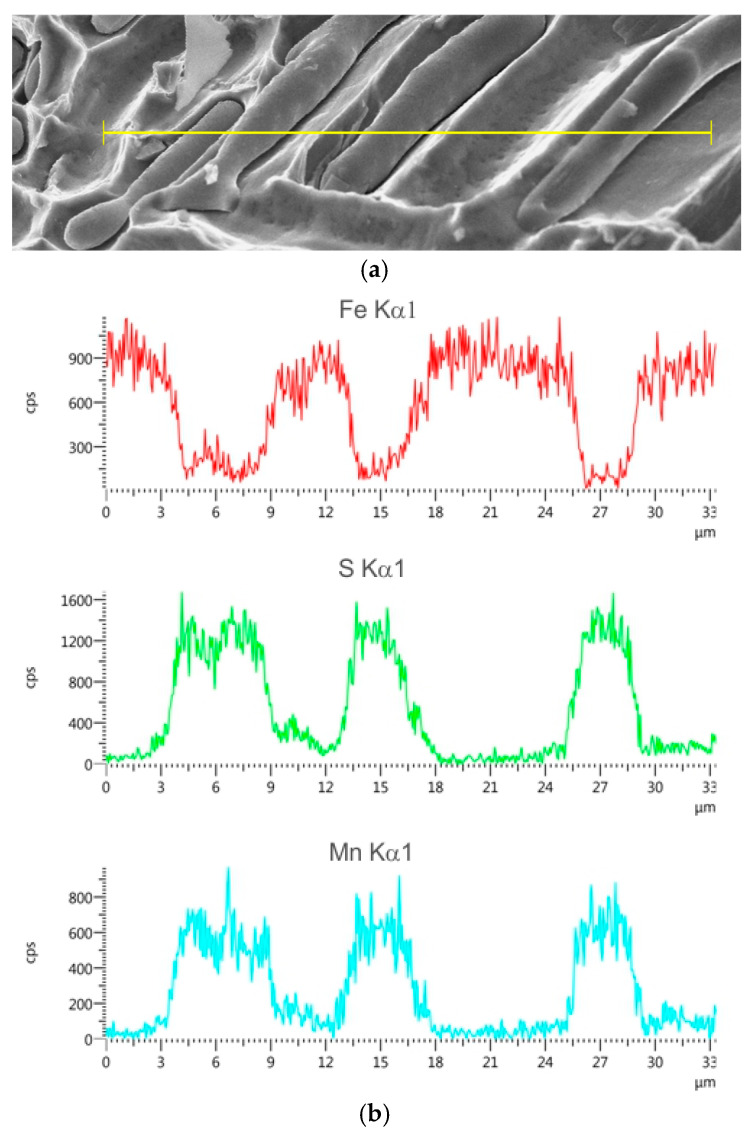
Morphologies (**a**) and line-scan mappings of MnS inclusions (**b**); scanning electron microscope.

**Figure 15:**
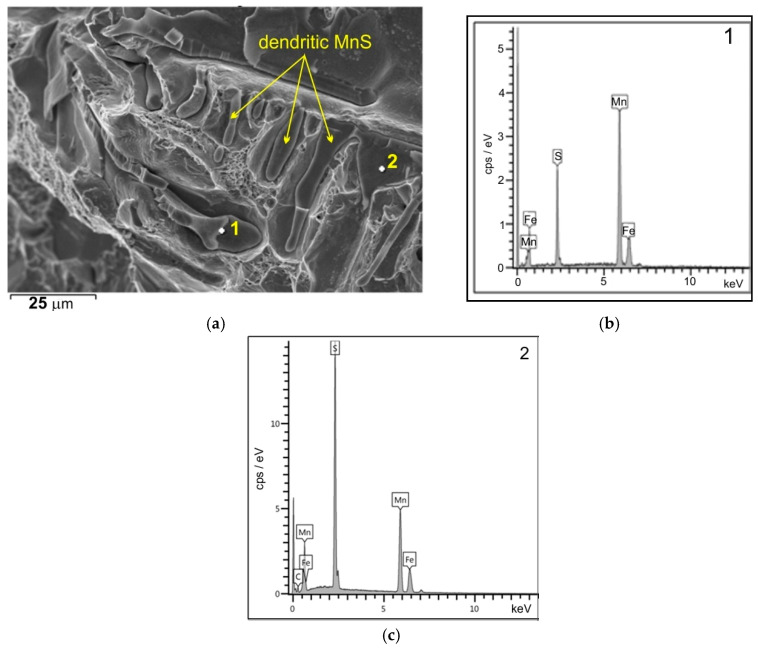
SEM images of a type II MnS inclusions on the fracture surface (**a**) from Figure 13a with the diffraction patterns from point 1 (**b**,**c**); scanning electron microscope.

The sulphide distribution pattern shown in Figure 13, Figure 14 and Figure 15 is characteristic of fractures observed in the central region (plate 4) and inner region (plate 7) of the wall cross-section at test temperatures of +20 °C and −10 °C. In these parts of the wall cross-section, numerous clusters of elongated inclusions are observed. Chemical analysis confirmed that these were the MnS sulphides of type II with dendritic morphology (Figure 13 and Figure 15a), whose effect on fracture mode and impact energy is more unfavourable than of type I or type III inclusions [18,26]. A large number of MnS inclusions concentrated in one location (Figure 13a), with small distances between them, cause stress concentrations and may consequently lead to premature fracture. Similar dendritic MnS precipitates in steel are described in [27,28]. MnS sulphides of this type are formed at the beginning of the solidification because the crystallisation temperature of MnS is above 1600 °C. Their morphology is related to the sulphur content, supersaturation, and crystal growth rate [18,28]. As reported by [26,27,29], with the increasing S content or the increasing cooling rate, more dendritic precipitates are formed. As reported in the literature [18,22,29], eutectic sulphide inclusions of elongated shape, distributed along grain boundaries, are also present. As a rule, these are FeS inclusions formed in the last stage of the casting solidification process.

The surface of the specimens cut from plate 8 (inner side, see Figure 1) exhibited visible cracks. As a result, their impact energy values were non-uniform, ranging from 6.8 to 29 J. In some cases, fracture did not initiate at the notch location, indicating irregular crack propagation behavior. This shows that the surface–crack network, induced by thermal fatigue, influences the impact strength of the examined cast steel.

### 3.4. Tensile Strength

To determine the variations in the mechanical properties across the cross-section of the slag ladle wall, uniaxial tensile tests were conducted. Based on prior microstructural examinations, hardness measurements, and impact energy tests, samples from plates 1, 2, 4, 5, and 7 were selected for testing.

**Figure 16:**
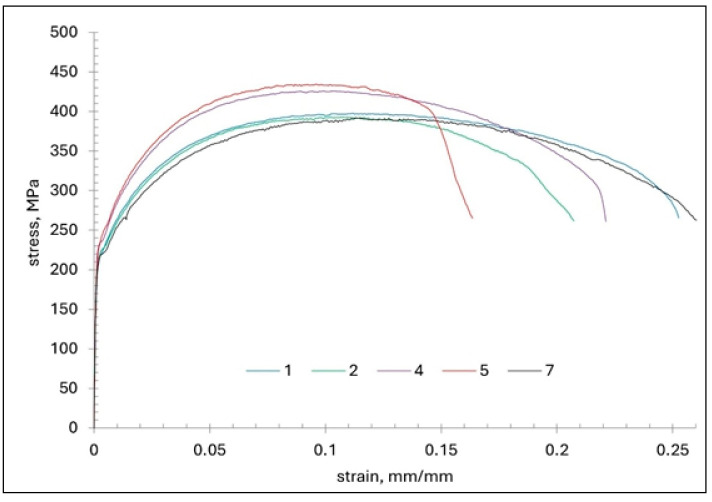
Selected tensile stress–strain curves.

**Figure 17:**
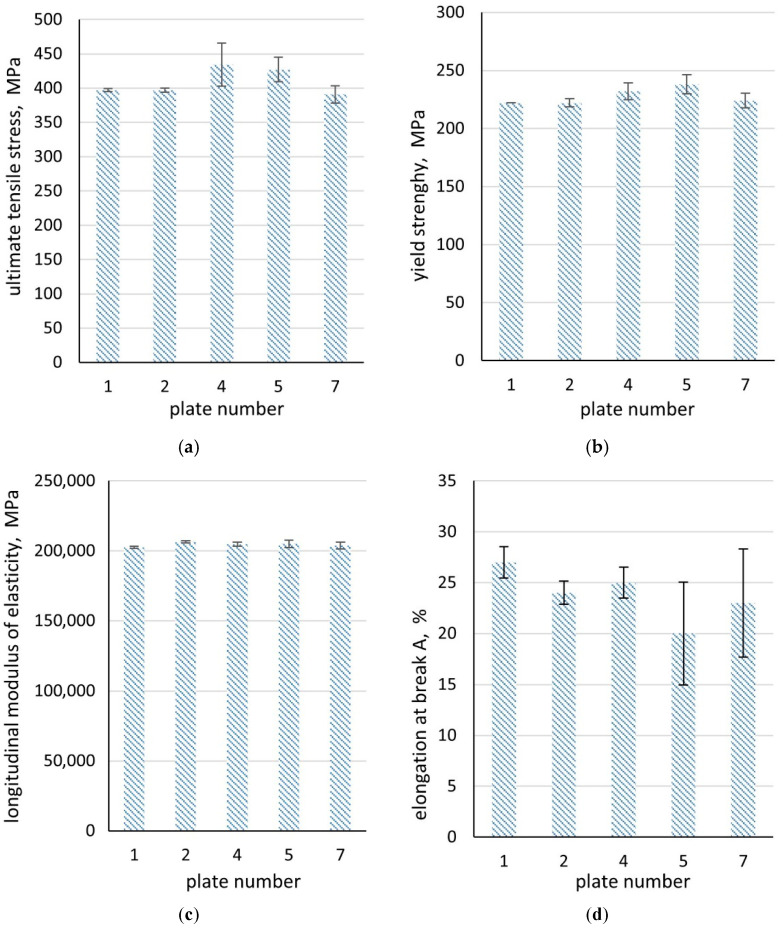
Distribution of mechanical properties in microscopic samples with a designated standard deviation: UTS (**a**), YS (**b**), longitudinal modulus of elasticity (**c**), and EL (**d**).

Three micro-specimens, extracted from selected macroscopic (impact) specimens, were subjected to tensile testing. The stress–strain curves for each examined section are presented in Figure 16. Figure 17 and Table 3 summarise the mean results obtained from the static tensile test.

**Figure 18:**
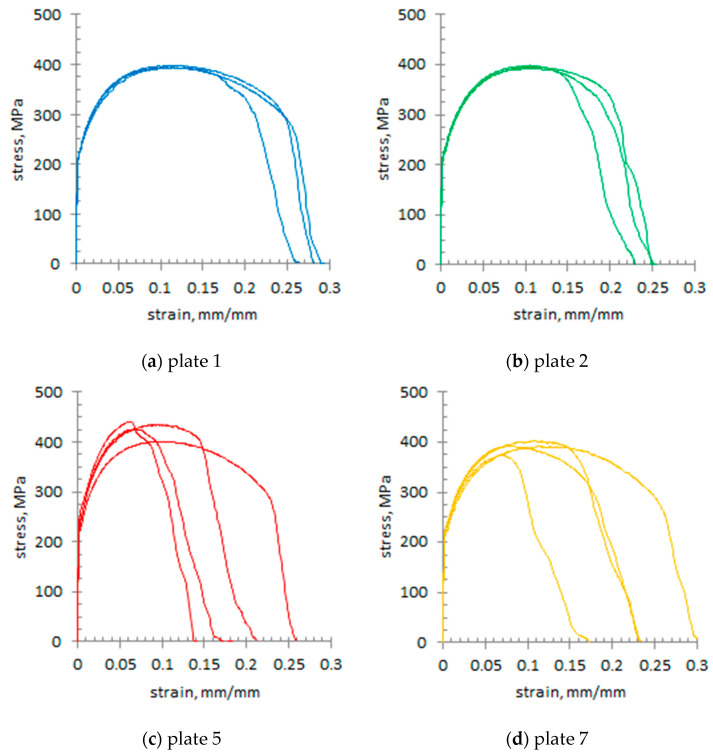
Stress–strain curves compared for plates 1 and 2 (**a**,**b**) and for plates 5 and 7 (**c**,**d**).

From the analysis of the stress–strain curves (Figure 16) it follows that, in the outer sections (plates 1 and 2), the curves show a similar course, with only minimal deviation (they practically overlap). This observation is consistent with the averaged results presented in Figure 16 and Table 3. Regarding the stress–strain curves for the central section of the wall (plate 4), the characteristic values are the highest; however, the scatter of parameters (Figure 16, Table 3) is definitely greater than for plates 1 and 2, with the deviation in the UTS in plate 4 reaching 30.5 MPa. In this case, the increase in strength parameters is due to carbon segregation and an increased pearlite fraction, while the presence of dendritic sulphide inclusions accounts for the variability in the results obtained from the micro-specimens. In plates 5 and 7, cut from the wall, the stress–strain curve is characterised by high irregularity. The reason is probably the presence of microstructural discontinuities in the form of microcracks, combined with the presence of non-metallic inclusions. This may result in a sudden collapse of the characteristics obtained for these plates (Figure 16 and Figure 18).

In the case of the modulus of longitudinal elasticity, EL, the scatter of the results on the cross-section of the casting wall was relatively small.

A sudden collapse of the stress–strain characteristics and, consequently, a significantly lower elongation at failure may indicate the occurrence of microcracks on the sample cross-section, which, once the critical stress value was exceeded, rapidly grew in length. The highest values of unit elongation EL were obtained in plate 1.

In summary, the differences between the values of the UTS and YS parameters across the ladle wall cross-section were smaller than those for elongation, hardness, or impact energy. Nevertheless, the values were slightly lower than those recommended by the standard [1].

Our research confirmed the importance of steel-melting technology for massive, large-size castings with walls exceeding 100 mm. It highlights the significance of liquid steel refining and modification processes aimed at increasing the metallurgical purity of liquid steel. They can effectively contribute to improving the properties of castings made of cast steel by reducing the volume fraction of non-metallic inclusions. This was particularly noticeable in the reduction of impact energy below the minimum value at temperatures under 0 °C (winter conditions for slag ladles—transport from the hall to the slag storage area). Moreover, the impact energy of specimens machined from the inner region of the slag ladle wall fell below the minimum required value of 27 J.

Another important factor in massive castings made of cast steel is the segregation of elements that occurs in thick cross-sections of the casting walls. In the case of the slag ladle casting, segregation of carbon and harmful sulphur was found. This finding confirms the principle that, for important massive castings, the permissible values of sulphur and phosphorus should not exceed 0.02% [12].

## 4. Conclusions

In this study, the microstructure, mechanical properties, and stress–strain characteristics were examined in seven plates taken from the cross-section of the wall of a massive slag ladle casting made from G17Mn5 steel. The following conclusions were drawn:

- Segregation of carbon and sulphur occurred across the wall cross-section between the outer and inner areas and the wall centre, where the highest C and S contents were measured. This segregation led to changes in the microstructure. Pearlite dominated in the centre of the wall, while ferrite with pearlite lakes prevailed in the outer and inner layers. Numerous MnS inclusions, mainly type II with dendritic morphology, were also observed. Only on the inner side of the ladle wall, at the surface after previous grinding, was the presence of a network of cracks reaching deep into the wall observed. These cracks resulted from the ladle’s operation under the cyclical thermal loading and mechanical stresses induced by liquid slag.
- In the central part of the wall (plate 4), the hardness of the tested cast steel reached approximately 140 HBW10, whereas in the remaining plates (numbered 1,2,3,5,6,7—Figure 1) the average hardness ranged from 125 to 130 HBW10.
- The average values of the impact energy in plates 1–3 at the temperature of +20 °C exceeded 40 J, whereas in plates 5–7, it was approx. 30 J. The lowest values were obtained at the centre of the wall (plate 4), coinciding with the region of maximum hardness. A similar trend in impact energy across the wall cross-section was obtained at 0 °C; however, the values did not exceed 20 J for plates 2–7, while plate 1 reached 22 J. The low impact energy values at the test temperature of −10 °C are worth noting. The values of the impact energy at both 0 °C and −10 °C is below the minimum required value of 27 J in the Charpy test.
- Changes in yield strength (YS) and ultimate tensile strength (UTS) across the ladle wall cross-section were found to correlate with changes in microstructure.
- The segregation of carbon and sulphur in the ladle wall cross-section was observed to have a significant effect on the strength characteristics, demonstrated, among others factors, by a markedly grater scatter of strength parameters in the wall centre (plate 4) compared with the outer plates (numbers 1 and 2) and inner plate (number 7). These variations may be attributed to the irregular distribution of non-metallic inclusions and the occurrence of microcracks.
- A high scatter in the elongation (EL) values of the tested material was observed in the inner region of the ladle wall cross-section (plates 5 and 7), which may be due to the presence of microcracks in the ladle wall.
- In the production of massive cast steel castings, the melting technology, particularly the refining and modification of molten steel, is a key factor influencing the final properties, primarily by enhancing purity.

## Figures and Tables

**Table 1:** Chemical composition of the tested cast steel.

**Cast steel** **G17Mn5** **(1.1131), [1]**	**C**	**Si**	**Mn**	**P**	**S**	**Cr**	**Al**	**Other**
	**Content wt. %**							
	0.15 0.20	max. 0.60	1.00 1.60	max. 0.020	max. 0.020	<0.3		<0.3% Cu <0.4% Ni
Melt	0.19	0.45	1.07	0.016	0.008	0.13	0.014	0.06% Cu 0.08% Ni

**Table 2:** Inclusion volume fraction and histogram of their size distribution.

Plate No.	Vol Fraction, %	<1 µm	1–10 µm	10–100 µm	>100 µm
1	0.26	873	689	276	28
2	0.20	227	125	231	32
3	0.22	888	519	288	22
4	0.30	392	224	243	44
5	0.60	156	132	308	118
6	0.20	352	179	212	25
7	0.20	138	199	184	20

**Table 3:** Averaged mechanical properties of macro-specimens determined from experimental tests.

Parameter	Number of Plate on the Casting Wall Cross-Section				
	1	2	4	5	7
UTS, MPa	397 ± 2	397 ± 2.5	434 ± 30.5	427 ± 20	391 ± 15
YS, MPa	222 ± 0	222 ± 3	232 ± 7	238 ± 8.5	224 ± 6
E, MPa	202,572 ± 767.5	206,357 ± 889.5	204,891 ± 1271	204,965 ± 3022.5	203,785 ± 2790.5
EL, %	27 ± 1.5	24 ± 1	25 ± 1	20 ± 5.5	23 ± 6.5

## Data Availability

Data is contained within the article. Further inquiries can be directed to the corresponding author.
